# Quantification of Oligonucleotides Using Tandem Mass Spectrometry with Isobaric Internal Standards

**DOI:** 10.3390/ijms241914691

**Published:** 2023-09-28

**Authors:** Christopher Gawlig, Güngör Hanci, Michael Rühl

**Affiliations:** BioSpring GmbH, Alt Fechenheim 34, 60386 Frankfurt am Main, Germany; gawlig@biospring.de (C.G.); hanci@biospring.de (G.H.)

**Keywords:** oligonucleotides, HPLC-MS/MS, isobaric standard, internal standard, bioanalytic, multiple reaction monitoring, fragmentation reaction, method validation, biological matrix

## Abstract

In recent years, oligonucleotides have become more important in research, drug approvals and medical therapies. Due to this growing interest in pharmaceutical applications, it is essential to develop reliable analytical methods for this substance class. In this work, we present a quantification method using liquid chromatography coupled with tandem mass spectrometry by applying an isobaric oligonucleotide standard. In addition to a proof of principle, we perform a method qualification to assess its readiness for validation according to ICH Q2 guidelines. In addition to good linearity, sensitivity, accuracy and recovery, the method showed no significant matrix effects. Furthermore, we demonstrated the application of the method by applying the quantification in a biological matrix, as well as an exemplary degradation of an oligonucleotide in bovine plasma.

## 1. Introduction

Oligonucleotide therapeutics have emerged as a promising class of drugs in recent years, with significant progress made in preclinical and clinical studies [1,2,3,4]. These therapeutics hold great potential for targeted treatments of various diseases [5,6,7] and come with different mechanisms of action like silencing RNA (siRNA), antisense oligonucleotide (ASO) and aptamers, or are used for gene therapy in combination with the CRISPR−Cas system [8,9]. In order to ensure the efficacy and safety of oligonucleotide therapeutics, (pre)clinical studies are performed. For the pharmacokinetic evaluation of the compounds, an accurate quantification to determine their concentration is crucial. The state-of-the-art methods for oligonucleotide quantification involve high-performance liquid chromatography (HPLC) coupled with UV [10], mass spectrometry (MS) [11] or fluorescent detectors [12]. Other commonly used methods include polymerase chain reaction (PCR) [13], capillary electrophoresis [14], fluorescent dye assays [15,16] and hybridization enzyme-linked immunosorbent assay (hELISA) [17,18]. The use of mass spectrometry, on the one hand, provides the opportunity to ensure the identity of the molecule but requires careful sample preparation, and on the other hand, avoids matrix effects and ion suppression [19]. A commonly used procedure is the use of stable isotope-labeled internal standards to quantify the compound of interest [20,21]. However, a gap exists when it comes to quantifying oligonucleotides using mass spectrometry compared to protein and peptides. While in the field of protein and peptide studies, MS/MS (tandem mass spectrometry) techniques for quantification with isobaric tags [22] such as tandem mass tags (TMTs) [23] are well described, a comparable approach is not yet readily available for oligonucleotides [24].

To address this gap, our study aimed to develop a method for oligonucleotide quantification using MS/MS with isobaric internal standards. The rationale for employing isobaric internal standards lies in their ability to produce distinct fragment ions that can be used for accurate quantification. Unlike precursor selection, where no significant differences might be present, the differentiation in fragment ion intensities allows for highly selective quantification of the molecule of interest.

Our approach involves the use of liquid chromatography coupled with tandem mass spectrometry (LC-MS/MS) to quantify oligonucleotides. Specifically, we utilize multiple reaction monitoring (MRM) of a single precursor ion and two distinct daughter ions for quantification. This approach serves as a proof of principle for accurate quantification and is demonstrated using a synthetic oligonucleotide and its isobaric internal standard. Here, we show the use of tandem-mass-spectrometry-based quantification, assess the main parameters as required by the ICH Q2 guidelines [25] and demonstrate the applicability for bioanalytical evaluation by extracting the compound from bovine plasma, as well as investigate the concentration of the oligonucleotide of interest over 192 h in plasma.

## 2. Results

### 2.1. Proof of Principle

**Figure 1:**
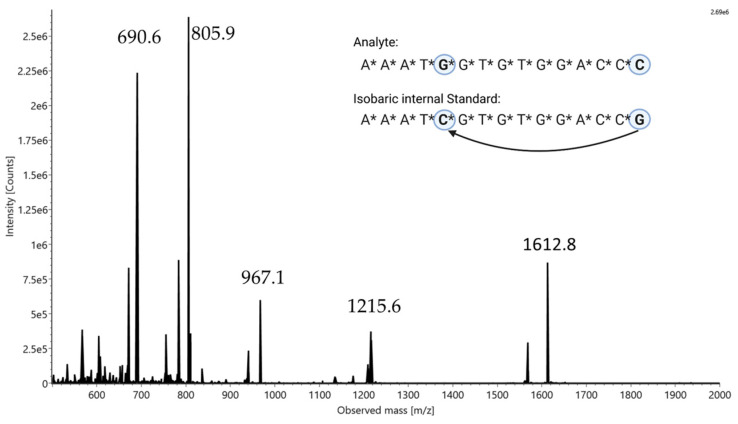
Sequences of isobaric oligonucleotide pair with highlighted base swaps (in the insert on the top right) and associated identical mass spectrum with labeled masses of charge states.

For the proof of principle of our described MS/MS quantification methodology, we use an isobaric oligonucleotide pair whose sequences are shown in Figure 1. These consist of a pair of 15-mers in which a base swap was performed at the 5th and 15th positions in the 5′ to 3′ direction. To ensure increased resistance and robustness to nucleases in a biological medium, both sequences are fully phosphorothiolated. The method described below is not limited by oligonucleotide modifications, as MS/MS quantification was also performed with non-phosphorothiolated isobaric oligonucleotides. For clarity, these experiments are listed in the Supplementary Materials (Figures S1–S11 and Tables S1–S16).

**Figure 2:**
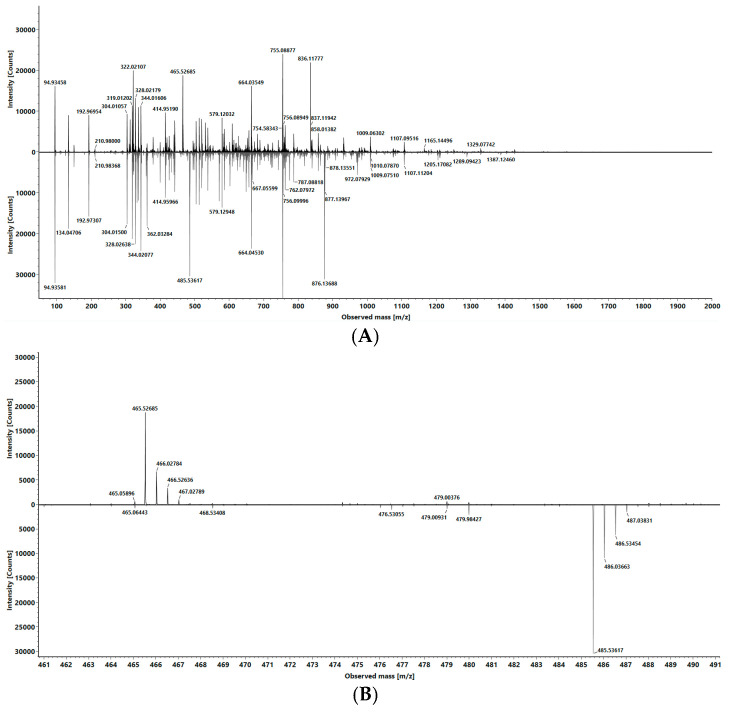
(**A**) MS/MS spectra of both isobaric oligonucleotides with precursor 690.52 *m*/*z*, comparative presentation; upper panel analyte, lower panel internal standard (IS). (**B**) Zoom of (**A**) focusing on the relevant mass range with the distinct fragment ions; upper panel analyte with 465.52 *m*/*z*, lower panel IS with 485.52 *m*/*z*.

Due to the isobaric character caused by the identical mass of the compounds, a mass spectrometric analysis of both sequences resulted in an indistinguishable spectrum (see Figure 1). In this spectrum, the different charge states of the oligonucleotide pair in the range from z = 3 (1612.8 *m*/*z*) to z = 7 (690.6 *m*/*z*) can be seen within the mass range of 500–2000 Da. All other unlabeled signals in the spectrum belong to synthesis impurities or shortmer by-products, but they are not a disturbance factor for the described method and will not be treated further. By selecting one of the charge states as a precursor for CID tandem MS analysis, it is possible to fragment both compounds without changing the selected precursor. The fragmentation of single molecules shows fragment ions that are specific to the respective molecule. In Figure 2, the fragment ion spectra for both isobaric oligonucleotides are depicted, which exhibit a few unique fragment ions. By selecting a uniquely assignable pair of those fragments, it is now possible to perform quantification according to the internal standard principle. In the case of our isobaric pair, the signal with the mass of 690.52 *m*/*z* (z = 6) was defined as the precursor ion; however, other charge states can be used equivalently (see Supplemental Figure S1). Nevertheless, a sufficiently high intensity should be ensured. Due to the different sequences of the last three nucleotides of the 3′ terminus, two unique fragment ions could be detected (Figure 2): 465.54 *m*/*z* as a w_3_ fragment ion with z = 2 from *C*C*C (McLuckey nomenclature [26]) and 485.54 *m*/*z* as a w_3_ fragment ion with z = 2 from *C*C*G. To ensure the formation of unique fragment ions, it is important to carry out the base swap with sufficient distance, for example, to the 3′ terminus. If the exchanged bases are too close to each other, indistinguishable isobaric fragment ions could form, making quantification impossible.

By defining the *C*C*C fragment (465.54 *m*/*z*) as analyte and the *C*C*G fragment as internal standard, it is possible to create a calibration line by preparing a dilution series of the “analyte” and adding a constant concentration of the “internal standard” and calculating the ratio of the fragment intensities. Therefore, our isobaric oligonucleotides will be called “analyte” and “isobaric standard (IS)” henceforth. To calculate the ratio between the analyte and isobaric standard for quantitation, we first employed a direct comparison of fragment ion intensities, but we were also able to utilize multiple reaction monitoring (MRM) area ratios for quantitative analysis. Depending on the software used, quantification can be performed using intensity ratios and the areas of the multiple reaction monitoring transitions 692.52 → 465.54 *m*/*z* and 690.52 → 485.54 *m*/*z*. For this purpose, the area ratio of the MRM areas of the two isolated fragment ions was used instead of the direct signal intensity (see Figure 2).

### 2.2. Qualification of Method Using ICH Q2 Guidelines

To optimize our method, we evaluated the optimal MS conditions by performing design of experiments (DoE) [27] for capillary voltage, sample cone voltage, source temperature, desolvation temperature and desolvation gas flow (see Section 4). The developed method was qualified to investigate its readiness for validation according to the guidelines on validation of analytical procedures from the European Medicines Agency (ICH Q2(R2) guidelines, 2018) [25]. We evaluated the method’s selectivity, linearity, sensitivity, precision, accuracy, recovery and matrix effects. For these assays, we diluted our analyte in bovine plasma as a biological medium and then extracted it for MS/MS quantification.

#### 2.2.1. Linearity

**Figure 3:**
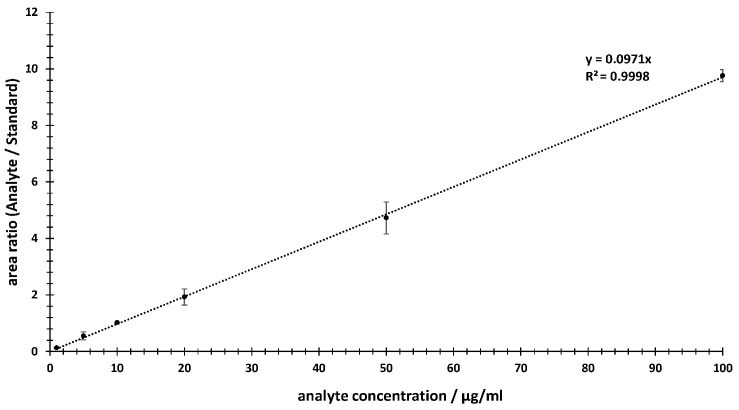
Calibration line from 1 µg/mL to 100 µg/mL via relative TIC areas of MRM experiments using the area ratios of the analyte compared to the internal standard.

For the linearity assessment, a calibration range was selected from 1 μg/mL to 100 μg/mL. To cover this range, six concentrations (1, 5, 10, 20, 50 and 100 μg/mL) of the analyte were prepared. Additionally, a final concentration of 10 µg/mL of internal standard was added per sample. The calibration curve was plotted using the peak area ratios of the analyte/IS vs. the concentrations of the dilution series within the calibration range (see Figure 3).

#### 2.2.2. Sensitivity

To determine sensitivity, a calibration line was measured with descending concentrations starting at 1 µg/mL. An LLOQ of 1 ng/mL of the analyte was found, at which linearity was present and precision was in the range of ±20%.

#### 2.2.3. Selectivity

**Figure 4:**
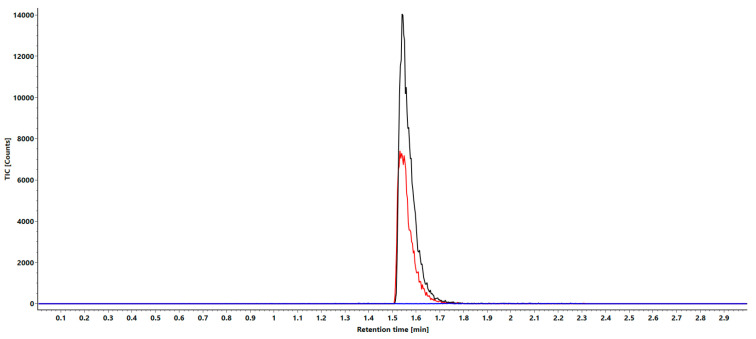
MRM overlays of analyte (black), standard (red) and bovine plasma (blue) with the significant channels 465 *m*/*z* for the analyte and 485 *m*/*z* for the standard.

In addition to the specificity discussed in the proof of principle section, the selectivity was confirmed by the absence of any peaks in the close proximity in terms of the mass of the analyte and IS in comparison to an injected blank bovine plasma sample. For this purpose, a solution of the LLOQ with 1 ng/mL of the analyte and IS, as well as an extracted solution of bovine plasma were fragmented and the mass ranges of 465.52 *m*/*z* and 485.52 *m*/*z* were compared. In addition to MS/MS fragmentation, MRM measurement was also performed with the two isolated target fragments. In Figure 4, the overlay of both ranges is depicted.

#### 2.2.4. Accuracy, Precision and Analyte Recoveries

To confirm the accuracy and recovery performance of the method, two analysis samples were prepared at 7 µg/mL and 25 µg/mL and then quantified in the calibration range of 1–100 µg/mL. For this purpose, samples and calibration standards were extracted from water and three replicates (*n* = 3) were measured over three days. Furthermore, the recoveries of our test analytes were calculated. Due to the use of an internal standard, a possible matrix effect arising from the extraction can usually be neglected in such investigations. To confirm this, the influences of extraction on the calibration standards and analytes were investigated. For this purpose, two calibration lines were prepared. One was untreated, with calibration standards prepared and extracted from nuclease-free water, and the other extractedfrom bovine plasma (see Section 4.2.2). The two analyte samples were also, like the calibration standards, both extracted from water and from bovine plasma to examine the impact on the recovery. For clarity, the extractions are presented and discussed separately.

(a) Calibration standards and samples from water extraction

The calibration standards and the analytical samples were prepared in nuclease-free water and subjected to a water extraction procedure before measurement (see Section 4.2.2). Average deviations of 5% at 7 µg/mL and 4% at 25 µg/mL were determined for the two analytes. This resulted in an average accuracy and recovery of over 95% for both samples. The precision of the method was expressed as the coefficient of variation (CV, %) and showed an average deviation of 9% between the individual values.

(b) Calibration standards and samples of non- and bovine plasma extract

**Figure 5:**
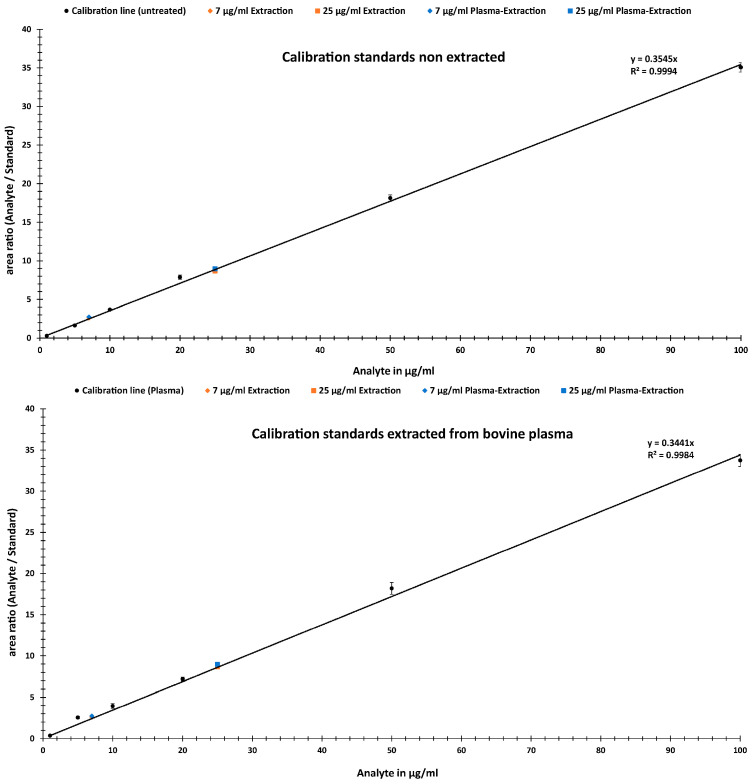
Calibration lines in the range of 1 to 100 µg/mL from non-extracted (**upper**) vs. bovine-plasma-extracted (**lower**) standards with added test analytical samples from water and bovine plasma extraction.

Figure 5 compares the two calibration curves. The upper graph was generated from non-extracted standards and the lower from bovine-plasma-extracted standards. Table 1 lists the recoveries for analyte analysis, as well as a comparison between plasma extraction and untreated samples. The mean values of the percentage error are ~7% for 7 µg/mL and ~2% for 25 µg/mL for bovine plasma and water extraction (see Table 1). Here, similarly, the accuracy has an average of 95%. The precision of the method was expressed as the coefficient of variation (CV, %) and was found to be around ±8% for both analytes and extractions.

### 2.3. Short-Term Biofluid Stability Study

**Figure 6:**
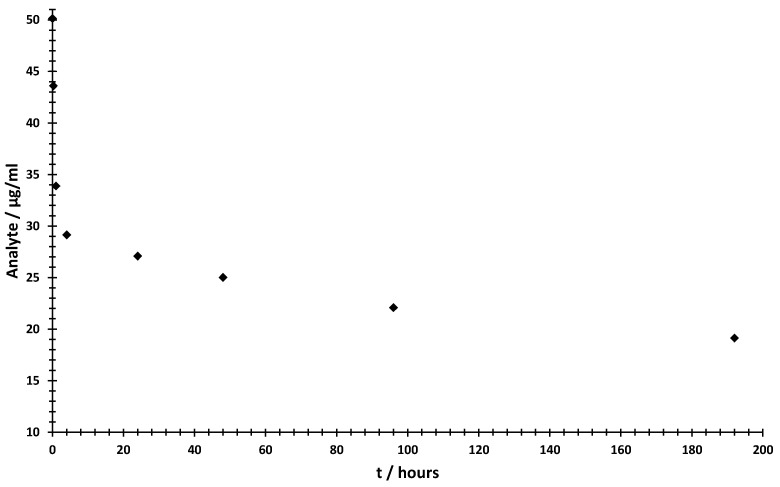
Time-dependent degradation in bovine plasma, as determined by MS/MS quantification, shows a fast degradation in the first 4 h and a slow degradation in the time up to 192 h. All data points represent the mean value ± SD (three biological replicates, nine measurements per data point).

To demonstrate an application for the method, an in vitro study using bovine plasma was performed to illustrate the metabolic degradation of the analyte. For this purpose, a concentration of 50 µg/mL of the analyte was incubated in bovine plasma at 37 °C for eight days. The sample collection time points were set at t = 0 min, 15 min, 1 h, 4 h, 24 h, 48 h, 96 h and 192 h. With this study, we wanted to simulate a pharmacokinetic study where samples are drawn at different time points. From previous studies, we know that plasma can degrade oligonucleotides at different positions [28]. We were interested in the concentration of the main compound. Figure 6 shows how the analyte degrades over time only by being stored in plasma at 37 °C.

## 3. Discussion

### 3.1. Linearity

Based on the calibration curve generated, the linearity in the selected concentration range of 1–100 µg/mL meets the criteria in which the obtained test results are directly proportional to the concentration (or amount) of analyte in the sample. In Figure 3, a linear behavior can be observed, with a Pearson correlation factor (R^2^) of 0.9998, a y-intercept of 0 and a slope of the regression line of 0.0971. In addition, the deviations of the individual data points were provided. These were within a range of 2–20%.

### 3.2. Sensitivity

The sensitivity was determined by the lowest amount of analyte in a sample, which requires a suitable precision and linearity. The experimental results obtained according to these criteria produced an LLOQ of 1 ng/mL. Linearity was assessed using LLOQ, yielding a Pearson correlation factor of R^2^ = 0.9955. Precession at the LLOQ was within the range of +20%, acceptable according to ICH Q2 guidelines [25]. Based on the literature, the quantification limit of 1 ng/mL was within the usual range of 0.5–50 ng/mL for oligonucleotides in the length range for chromatographic quantification methods [29].

### 3.3. Selectivity

This shows that in both fragment ranges, there was no overlapping of signals of the other isobaric oligonucleotide that would lead to a misinterpretation in the quantification. Additionally, the comparison with the spectrum of a blank bovine plasma showed no signals of sufficient intensity in either region to interfere with quantification (Figure 4).

### 3.4. Accuracy, Precision and Matrix Effect Evaluation

High accuracies of at least 88–96% at 7 µg/mL and 96–99% at 25 µg/mL were achieved in the assay analytes (Table 1). The recoveries were, on average, 98–111%. Compared to published chromatographic quantification methods, with recoveries of 80–106%, they are comparable or slightly better [30,31,32,33]. By comparing standards extracted from different media for calibration line generation and analytes, it can be determined whether matrix effects can falsify the quantification results. As expected, in the comparison of the accuracy in Table 1, no significant influence of the medium or the extraction itself was observed for either of the two analytes or the ratio between the analyte and internal standard. The deviations of extraction from water and bovine plasma at 7 µg/mL were approximately 6%. The calibration standards were also extracted from water and bovine plasma and the analytical results were compared. Maximum deviations of 3% in accuracy were found. Thus, the extraction of the calibration standards also showed no major influence on the accuracy of the analysis. This was to be expected since for the calibration, the TIC area ratios of the isobaric pair are important for the quantification and are not influenced by any losses due to the medium and the extraction process.

### 3.5. Short-Term Biofluid Stability Study

By determining the time-dependent concentration of the analyte in bovine plasma, we can demonstrate the specific application and functionality of the MS/MS quantification method. Figure 6 shows two interesting areas with different conversion rates. The highest rate occurred in the first 4 h, with a degradation rate of 87.5 µg/mL·min. After this initial phase of fast degradation, the substrate conversion slowed down to 0.887 ng/mL·min. This could be due to inhibition of the respective enzymes or the degradation or inactivation of the plasma in the experimental setup. Nevertheless, we could show that we were able to investigate the concentration over a short period of time with high accuracy. The method would therefore be suitable for pharmacokinetic investigations in early drug development.

## 4. Materials and Methods

### 4.1. Materials

All oligonucleotides and isobaric internal standards were synthesized in-house by Biospring GmbH (Frankfurt, Germany) via solid-phase synthesis according to Beaucage et al. [34]. The used oligonucleotides are listed in Table 2. 1,1,1,3,3,3-Hexafluoroisopropanol ≥99.8% was purchased from Biosolve (Valkenswaard, Netherlands). Bovine plasma was purchased from Biowest (Nuaillé, France). Microreaction tubes were purchased from Eppendorf (Hamburg, Germany). Ammonium acetate, LC-MS-grade methanol (MeOH) and triethylamine (puriss. plus) were purchased from Merck KGa (Darmstadt, Germany). OTX Loading-Lysis buffer was purchased from Phenomenex (Aschaffenburg, Germany). Acetic acid, HPLC-grade acetonitrile, ammonia, ammonium bicarbonate and tetrahydrofuran were purchased from Carl-Roth (Karlsruhe, Germany). Phosphate-buffered saline (PBS), recombinant Proteinase K and nuclease-free water were purchased from Thermo Scientific (Dreieich, Germany). Oasis WAX 1 cc Vac Cartridge, Total Recovery Vials and the Acquity UPLC BEH C18 Column, 130 Å, 1.7 µm, 2.1 mm x 50 mm column were purchased from Waters (Eschborn, Germany).

### 4.2. Sample Preparation

#### 4.2.1. Preparation of Testing Analytes and Calibration Standards

Calibration solutions and testing analytes were prepared by diluting the analyte to the desired concentrations using nuclease-free water. After dilution, a set amount of IS was added to every dilution of the analyte. The used concentrations can be found in the respective results.

#### 4.2.2. Solid-Phase Extraction from Biological Medium

The solid-phase extraction was performed using Oasis SPE columns in combination with a vacuum manifold according to the manufacturer’s manual. The columns were flushed with methanol and equilibrated using 50 mM ammonium acetate pH 5.5. After equilibration, the sample was loaded onto the column and washed three times with 50 mM ammonium acetate mixed with acetonitrile (50:50, Vol:Vol). For sample elution, 100 mM ammonium bicarbonate pH 10.5 mixed with acetonitrile and THF (50:40:10, Vol:Vol:Vol) was used. The samples were dried in a vacuum centrifuge and either stored at −20 °C or reconstituted in eluent A and measured.

#### 4.2.3. Preparation of In Vitro Degradation Samples

The analyte was diluted to 50 µg/mL using bovine plasma at a volume of 3 mL. This master dilution was aliquoted to 24 samples at a volume of 90 µL each and the samples were incubated at 37 °C and 500 rpm. At determined time points, samples were removed from the thermoshaker in triplicate and frozen and stored at −70 °C. Once all samples were obtained, IS was added to a concentration of 10 µg/mL, and the samples were thawed with an equivalent volume of OTX Loading-Lysis buffer. Solid-phase extraction was performed according to Section 4.2.2.

### 4.3. HPLC-MS/MS Analysis

The LC flow rate was set to 0.3 mL/min. Eluent A consists of a buffer system with 7 mM TEA and 80 mM HFIP solution in water. Eluent B consists of a mixture of 50% Eluent A and 50% methanol. The injection volume was set to 2 μL with the column temperature at 60 °C. An Acquity UPLC BEH C18, 130 Å, 1.7 µm, 2.1 mm × 50 mm column was used for all measurements. Eluent B elution gradient was set as follows: 0–0.20 min (min) at 10%, 0.20–2.00 min from 10 to 100%, 2.00–2.30 min at 100% and 2.30–3.00 min from 100% to 10%. All ESI-MS measurements on the Vion IMS qTOF mass spectrometer were performed in negative ion mode. Calibration of MS data was achieved through constant infusion of leucine enkephalin calibration solution by Waters (SKU: 186006013) at a flow rate of 10 µL/min. The MS parameters were previously optimized in the design of the experimental procedure. The key parameters used were as follows: 3.2 kV capillary voltage, 40 V cone voltage and 100 V offset voltage. The source temperature was set to 80 °C with a desolvation temperature of 500 °C. The cone gas flow was set to 50 L/h and the desolvation gas flow to 800 L/h.

### 4.4. Data Analysis and Visualization

Data acquisition and processing were performed with UNIFI^TM^ (1.9.13.9 and 3.0.0.15). Design of experiments was performed using MODDE Pro Version 13 with a full-factorial model for data analysis. All measurements presented in this publication were reproduced at least three times and all data points were formed from the mean value of triplicate measurements. The conversions from the area ratios of the analyte and internal standards to the concentrations were performed by rearranging the straight-line equation of the calibration line:

$$concentration=\frac{area\;ratio(\frac{Analyte}{Internal\;Standard})}{regression\;line\;slope}$$

The graphical abstract was drawn using the online BioRender in its current version (August 2023).

## 5. Conclusions

Oligonucleotides are emerging new therapeutics, especially in the fields of gene therapy, inherited diseases and personalized medicine. For all new chemical entities used for therapy, a thorough characterization of the compound is inevitable. In particular, the quantitative analysis of oligonucleotides from biofluids and tissue is challenging. This study presents a robust and accurate method for oligonucleotide quantitation using mass spectrometry. As isobaric tags are not available for oligonucleotides and stable isotope labels are expensive, we developed a cost-efficient and innovative method to address this disparity. We have shown that the use of isobaric internal standards, in combination with MRM analysis, yielded good accuracy, recovery and linearity for the mid-concentration analytes (1 – 100 µg/mL) down to an LLOQ of 1 ng/mL. The use of the method for quantitation from biological fluids showed the applicability of the method for the intended purpose. Through a limited method qualification, we were also able to show that this quantification approach is ready for validation according to ICH Q2 guidelines.

## Figures and Tables

**Table 1:** Sample analyte recoveries and percentage deviations from water and bovine plasma extraction.

Extraction from	7 µg/mL ^1^	25 µg/mL ^1^	7 µg/mL ^2^	25 µg/mL ^2^
H_2_O	7.34 µg/mL ^1^	24.46 µg/mL ^1^	7.57 µg/mL ^2^	24.46 µg/mL ^2^
Δ(H_2_O)	4% ^1^	2% ^1^	7% ^2^	2% ^2^
bovine plasma	7.72 µg/mL ^1^	25.28 µg/mL ^1^	7.96 µg/mL ^2^	26.05 µg/mL ^2^
Δ(bovine plasma)	10% ^1^	1% ^1^	12% ^2^	4% ^2^

^1^ Calibration line, non-extracted. ^2^ Calibration line, extracted from bovine plasma.

**Table 2:** Isobaric oligonucleotide pairs used for MS/MS quantification. Asterisks show thiolations.

Isobaric Pair 1	Isobaric Pair 2 ^1^
A*A*A*T*G*G*T*G*T*G*G*A*C*C*C	AAATGGTGTGGACCC
A*A*A*T*C*G*T*G*T*G*G*A*C*C*G	AAATCGTGTGGACCG

^1^ Data in Supplementary Information.

## Data Availability

Data are contained within the article and Supplementary Materials.

## Supplementary Materials

The following supporting information can be downloaded at: https://www.mdpi.com/article/10.3390/ijms241914691/s1.
