# Supplementary material for: Quantification of Oligonucleotides Using Tandem Mass Spectrometry with Isobaric Internal Standards
Source: Int J Mol Sci. 2023 Sep 28;24(19):14691. doi: 10.3390/ijms241914691 (PMC10573027; doi:10.3390/ijms241914691)
Supplement: Supplementary file 1 [file ijms-24-14691-s001.zip › ijms-2606832-supplementary.pdf]

Supplemental Information

for

Quantification of oligonucleotides by isobaric internal standards using tandem mass spectrometry

by

Christopher Gawlig, Güngör Hanci and Michael Rühl

1. DoE parameter optimization ... 3
Table S1: Runs performed for intensity optimisation with respective results for ion at m/z = 690.6 ... 3
Figure S1: Contour plot of DOE results ... 5
2. MSMS Quantification of non-thiolated Oligonucleotides ... 6
Figure S2: Calibration curve of isobaric pair 2 via intensity. ... 6
Table S2: Raw data for creation of calibration curve in Figure S1 via signal intensity (An = analyte; IS = internal standard). ... 6
Figure S3: Calibration curve of isobaric pair 2 via MRM TIC area. ... 7
Table S3: Raw data for creation of calibration curve in Figure S2 via MRM TIC area (An = analyte; IS = internal standard). ... 7
3. Method Qualification – Linearity ... 8
Figure S4: Calibration curve of isobaric pair 1 via intensity. ... 8
Table S4: Raw data for creation of calibration curve in Figure S3 signal intensity (An = analyte; IS = internal standard). ... 8
Figure S5: Calibration curve of isobaric pair 1 via MRM TIC area. (see Figure 4) ... 9
Table S5: Raw data for creation of calibration curve in Figure S4 via MRM TIC area (An = analyte; IS = internal standard). ... 9
4. Method Qualification – Sensitivity ... 10
Figure S6: Sensitivity experiments of isobaric pair 1 analyte via MRM TIC area on a logarithmic scale. With LLOQ of 1 ng/ml. ... 10
Table S6: Raw data for sensitivity experiment in Figure S5 via MRM TIC area (An = analyte; IS = internal standard). ... 10
Table S6: Raw data for precision determination of LLOQ in Figure S5 via MRM TIC area (An = analyte; IS = internal standard) ... 10
5. Method Qualification – Accuracy & Precision ... 11
Figure S7: Calibration curve of isobaric pair 1 via MRM TIC area; non-extracted. (see Figure 5) ... 11
Figure S8: Calibration curve of isobaric pair 1 via MRM TIC area; extracted from bovine plasma. (see Figure 5) ... 11
Table S8: Raw data for non-extracted calibration curve in Figure S6 via MRM TIC area (An = analyte; IS = internal standard) ... 12
Table S9: Raw data for calibration curve extracted from bovine plasma in Figure S7 via MRM TIC area (An = analyte; IS = internal standard). ... 12
Table S10: Raw data for sample testing extracted from water in Figure S6 & S7 via MRM TIC area (An = analyte; IS = internal standard). ... 12
Table S11: Raw data for sample testing extracted from bovine plasma in Figure S6 & S7 via MRM TIC area (An = analyte; IS = internal standard). ... 12
Table S12: Raw data for precision calculation by triplicate replica from analyte sample testing ... 13
6. Method Qualification – Short term biofluid stability study ... 13
Table S13: Raw data for biofluid stability study in bovine plasma via MRM TIC area; set 1 (An = analyte; IS = internal standard) ... 13
Table S14: Raw data for biofluid stability study in bovine plasma via MRM TIC area; set 2 (An = analyte; IS = internal standard) ... 14

Table S15: Raw data for biofluid stability study in bovine plasma via MRM TIC area; set 3 (An = analyte; IS = internal standard).. 14

1. DoE parameter optimization

For optimal MS-parameters, we assessed the critical parameters influencing the intensity of one of the charge states of our molecule. For this purpose, we were using the software MODDE Version 13. We used the screening objective with full factorial design with interactions. The runs used to build the model are shown in supplementary table 1. Figure S1 shows the Contour plot of the DoE experiments calculated by MODDE software. Best results were considered for final method settings. See Material and methods. The optimizations were all performed with Precursor 690 m/z (z = 7). The optimizations were all performed with Precursor 690 m/z (z = 7). However, the use of other charge states is also possible and results in equivalent fragment spectra. As an example, a comparison of the MSMS spectra of precursor 690 m/z and 805 m/z was compared in Figure S1. Here, the fragments 465 m/z & 485 m/z relevant for quantification are clearly visible.

Table S1: Runs performed for intensity optimisation with respective results for ion at m/z = 690.6

Run order	Exp Name	Source temperature [°C]	Capillary voltage [kV]	Cone voltage [V]	Desolvation temperature [°C]	Desolvation gas flow [L/h]	Intensity of 690.6 m/z
1	N9	80	2.8	40	500	725	19825
2	N11	80	3.2	40	500	725	29093
3	N27	80	3.2	40	500	775	17335
4	N21	80	2.8	60	450	775	18026
5	N35	100	3.0	50	475	750	12513
6	N17	80	2.8	40	450	775	10839
7	N15	80	3.2	60	500	725	
8	N8	120	3.2	60	450	725	19488
9	N18	120	2.8	40	450	775	14946
10	N33	100	3.0	50	475	750	13748
11	N24	120	3.2	60	450	775	15600
12	N10	120	2.8	40	500	725	20146
13	N23	80	3.2	60	450	775	16407
14	N32	120	3.2	60	500	775	16391
15	N25	80	2.8	40	500	775	22271
16	N12	120	3.2	40	500	725	24479
17	N29	80	2.8	60	500	775	13354
18	N2	120	2.8	40	450	725	26737
19	N7	80	3.2	60	450	725	24079
20	N19	80	3.2	40	450	775	15007
21	N31	80	3.2	60	500	775	18303
22	N14	120	2.8	60	500	725	19481
23	N3	80	3.2	40	450	725	30323
24	N30	120	2.8	60	500	775	14311
25	N1	80	2.8	40	450	725	26263
26	N20	120	3.2	40	450	775	18005
27	N13	80	2.8	60	500	725	17320
28	N22	120	2.8	60	450	775	18053
29	N26	120	2.8	40	500	775	19403
30	N5	80	2.8	60	450	725	18336
31	N28	120	3.2	40	500	775	17444
32	N4	120	3.2	40	450	725	24337
33	N34	100	3.0	50	475	750	13365
34	N6	120	2.8	60	450	725	16409
35	N16	120	3.2	60	500	725	

A)

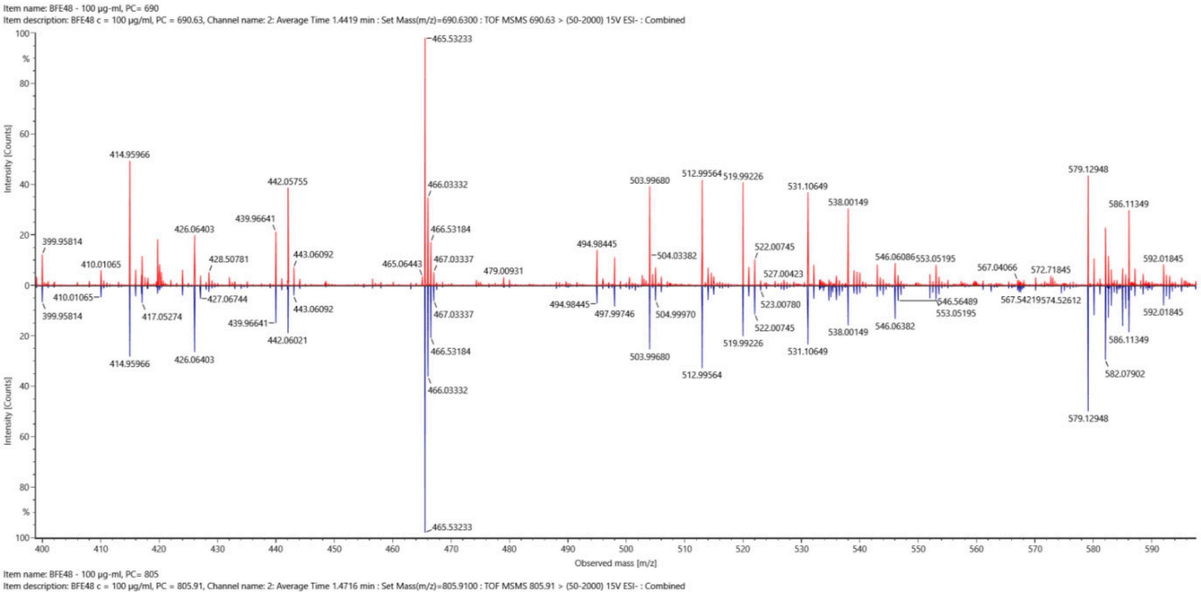

B)

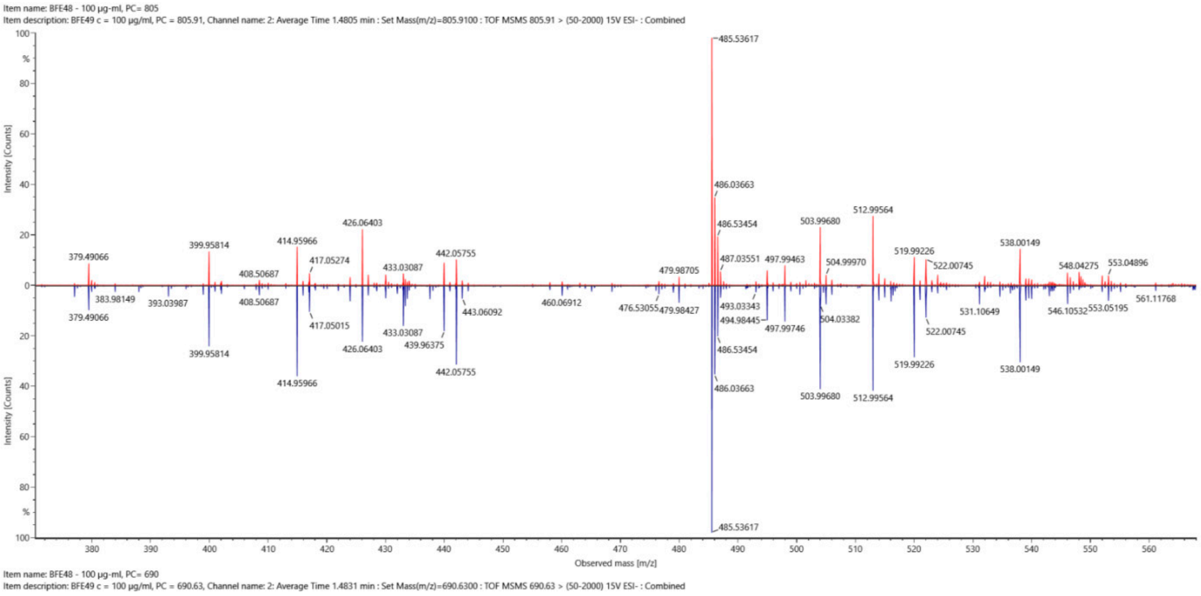

Figure S1: Comparison of the fragment spectra of the 690 m/z and 805 m/z precursors. **A**: Mass range with the fragment 465 m/z from precursor 805 m/z (top) and 690 m/z (bottom), which is relevant for quantification. **B**: Mass range with the fragment 485 m/z from Precursor 805 m/z (top) and 690 m/z (bottom) that is relevant for quantification.

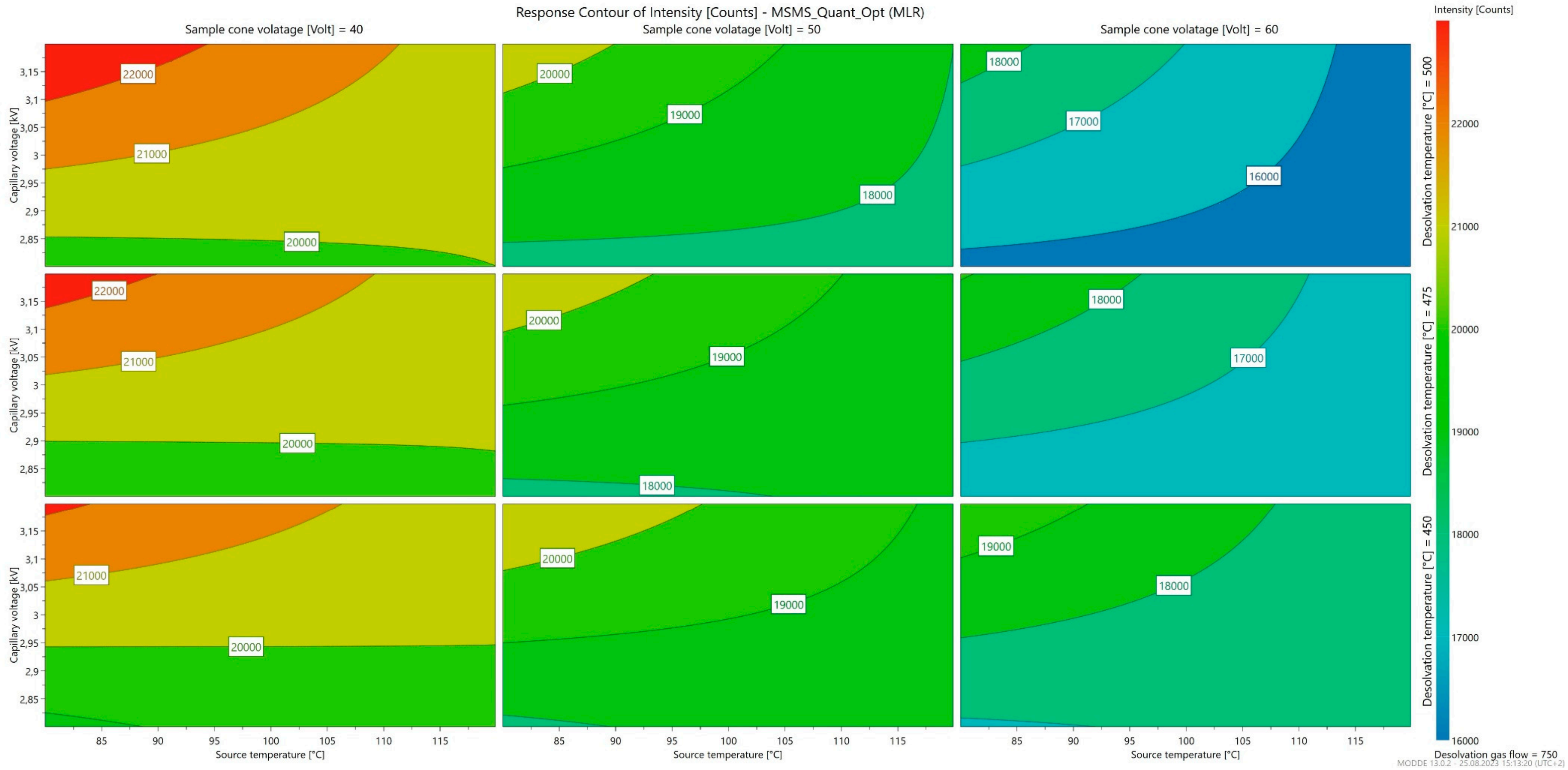

Figure S2: Contour plot of DOE results.

2. MSMS Quantification of non-thiolated Oligonucleotides

In addition to the isobaric pair used in the publication, proof of principle was also performed with a non-thiolated oligonucleotide pair (Chapter 4.1, Table 2). In this case, the MRM TIC area was calibrated via the signal intensity and the MRM TIC area. It was found that a linear trend line cannot be applied to the data points when calibrating via the intensity. Instead, the course can be described via a power equation. This can also be seen in the case of the thiolated oligonucleotide pair, which is why calibration via MRM TIC areas also appears to make more sense in this case.

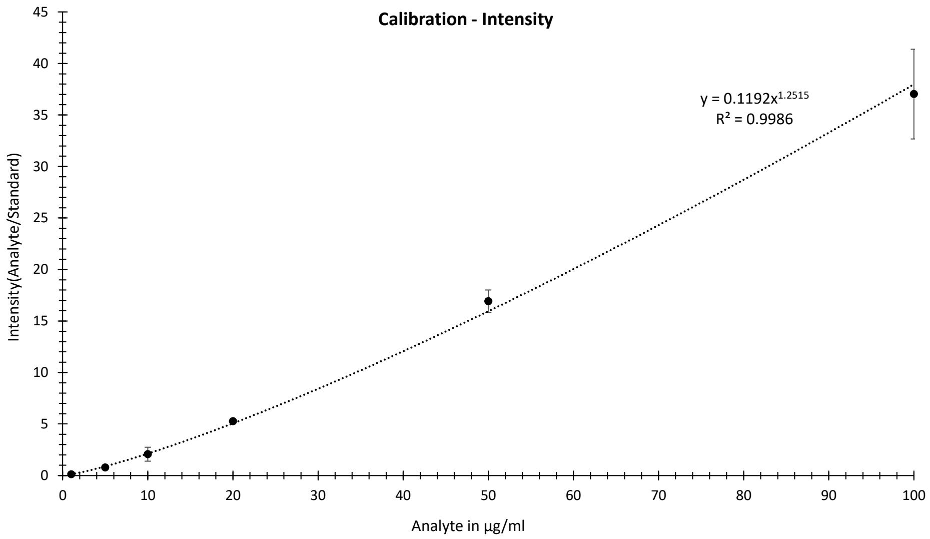

Figure S3: Calibration curve of isobaric pair 2 via intensity.

Table S2: Raw data for creation of calibration curve in Figure S1 via signal intensity (An = analyte; IS = internal standard).

Calibration I - Intensity								
Measurement	c(An)	I(An)	X̄(An)	I(IS)	X̄(IS)	ratio[I(An)/I(IS)]	X̄[I(An/I(IS)]	σ[I(An/I(IS)]
1		154057		1617497		0.095244072		
2		133743		1325702		0.100884663		
3	1 µg/ml	133849	135573	989243	1126962	0.13530447	0.126702593	0.027824794
4		127185		898843		0.141498571		
5		129031		803525		0.160581189		
1		693420		1005906		0.689348707		
2		730382		862749		0.846575308		
3	5 µg/ml	725998	715918.4	890747	900669	0.815044002	0.798185865	0.06409279
4		730693		867337		0.842455701		
5		699099		876607		0.79750561		
1		876607		955745		0.917197579		
2		1605261		688744		2.330707781		
3	10 µg/ml	1649151	1429500.4	654173	724830	2.520970752	2.073244697	0.678583397
4		1468108		720764		2.036877536		
5		1548375		604723		2.560469835		
1		2716210		521979		5.203676776		
2		2749185		515384		5.334245921		
3	20 µg/ml	2745403	2726256.6	511908	516120	5.363078913	5.28276988	0.068559113
4		2675771		505960		5.288503044		
5		2744714		525370		5.224344748		
1		5616577		324213		17.32372545		
2		5730654		312552		18.33504185		
3	50 µg/ml	5804163	5588423.6	375270	331428	15.46663202	16.92546552	1.080295373
4		5580308		341458		16.34258972		
5		5210416		303649		17.15933858		
1		6899815		196576		35.09998677		
2		6961989		209434		33.24192347		
3	100 µg/ml	7167270	6996595.2	179563	191035	39.91507159	37.03400166	4.359006821
4		7141847		212019		33.68493861		
5		6812055		157584		43.22808788		

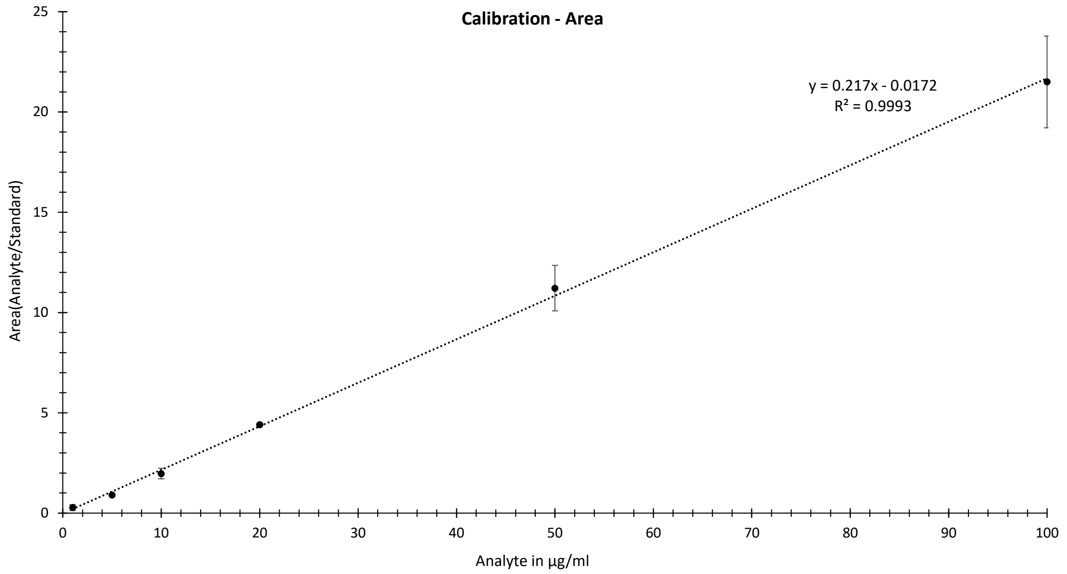

Figure S4: Calibration curve of isobaric pair 2 via MRM TIC area.

Table S3: Raw data for creation of calibration curve in Figure S2 via MRM TIC area (An = analyte; IS = internal standard).

Calibration I - Area								
Measurement	c(An)	A(An)	X̅(An)	A(IS)	X̅(IS)	ratio[A(An)/A(IS)]	X̅[A(An)/A(IS)]	σ[A(An)/A(IS)]
1	1 µg/ml	109182	104207.6	205609	446840	0.531017611	0.272369817	0.147154383
2		106338		648002		0.164101345		
3		105162		507913		0.20704727		
4		100292		451075		0.222339966		
5		100064		421601		0.237342891		
1	5 µg/ml	401332	411919.2	497189	456910	0.80720209	0.903747505	0.055320398
2		410811		452577		0.907715151		
3		419650		450533		0.931452302		
4		414047		444193		0.932133104		
5		413756		440056		0.940234879		
1	10 µg/ml	759033	772196.6	502451	399379	1.510660741	1.964864987	0.26400505
2		776828		397018		1.956656877		
3		784070		374830		2.091801617		
4		783000		366380		2.137125389		
5		758052		356214		2.128080311		
1	20 µg/ml	1314852	1320227	296480	300086	4.434875877	4.400322135	0.059931782
2		1323744		296061		4.471186681		
3		1314994		298234		4.409269232		
4		1311572		300075		4.370813963		
5		1335973		309578		4.315464923		
1	50 µg/ml	2598585	2640745.2	234620	237599	11.07571818	11.21233832	1.134267506
2		2656863		251891		10.54766943		
3		2678581		273823		9.7821622		
4		2633155		218454		12.05359023		
5		2636542		209207		12.60255154		
1	100 µg/ml	2858083	3052872.4	144820	142145	19.73541638	21.63515943	2.285727571
2		3054761		164088		18.61660207		
3		3120077		135443		23.03608898		
4		3122559		132795		23.5141308		
5		3108882		133580		23.27355892		

3. Method Qualification – Linearity

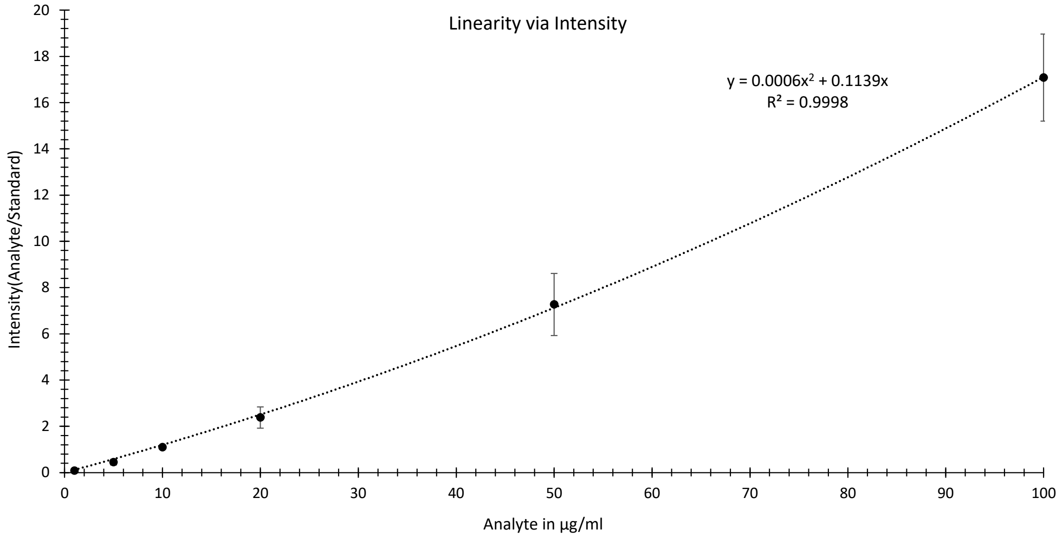

Figure S5: Calibration curve of isobaric pair 1 via intensity.

Table S4: Raw data for creation of calibration curve in Figure S3 signal intensity (An = analyte; IS = internal standard).

Calibration - Intensity								
Measurement	c(An/IS)	I(An)	X̅(An)	I(IS)	X̅(IS)	ratio[I(An)/I(IS)]	X̅[I(An)/I(IS)]	σ[I(An)/I(IS)])
1	1 µg/ml	359632	310617.4	17266	24091	0.048010188	0.078623605	0.019480198
2		293342		20974		0.07150016		
3		266859		22771		0.085329706		
4		252227		24081		0.095473522		
5		381027		35361		0.092804447		
1	5 µg/ml	351922	342928.2	88149	154251	0.250478799	0.448405117	0.12350485
2		305989		124616		0.40725647		
3		317342		169966		0.535592515		
4		388051		212191		0.546812146		
5		351337		176331		0.501885654		
1	10 µg/ml	524686	573733	550636	628575	1.049458152	1.096042124	0.04944966
2		542737		612089		1.127781964		
3		581392		660633		1.136295305		
4		589309		666868		1.131610072		
5		630541		652651		1.035065127		
1	20 µg/ml	614719	499098	1006443	1164925	1.637240756	2.381091934	0.462632854
2		488201		1087562		2.227693102		
3		485666		1274230		2.62367553		
4		454413		1255901		2.763787568		
5		452491		1200487		2.653062713		
1	50 µg/ml	369988	300981.6	1966505	2141802	5.315050758	7.26751094	1.341390023
2		313725		2070741		6.600497251		
3		287541		2163250		7.523274942		
4		265523		2297043		8.651013283		
5		268131		2211469		8.247718466		
1	100 µg/ml	180932	162403	2621674	2757273	14.48983043	17.08066615	1.884692985
2		167111		2651840		15.86873396		
3		155860		2744157		17.60655075		
4		155327		2975424		19.15587116		
5		152785		2793268		18.28234447		

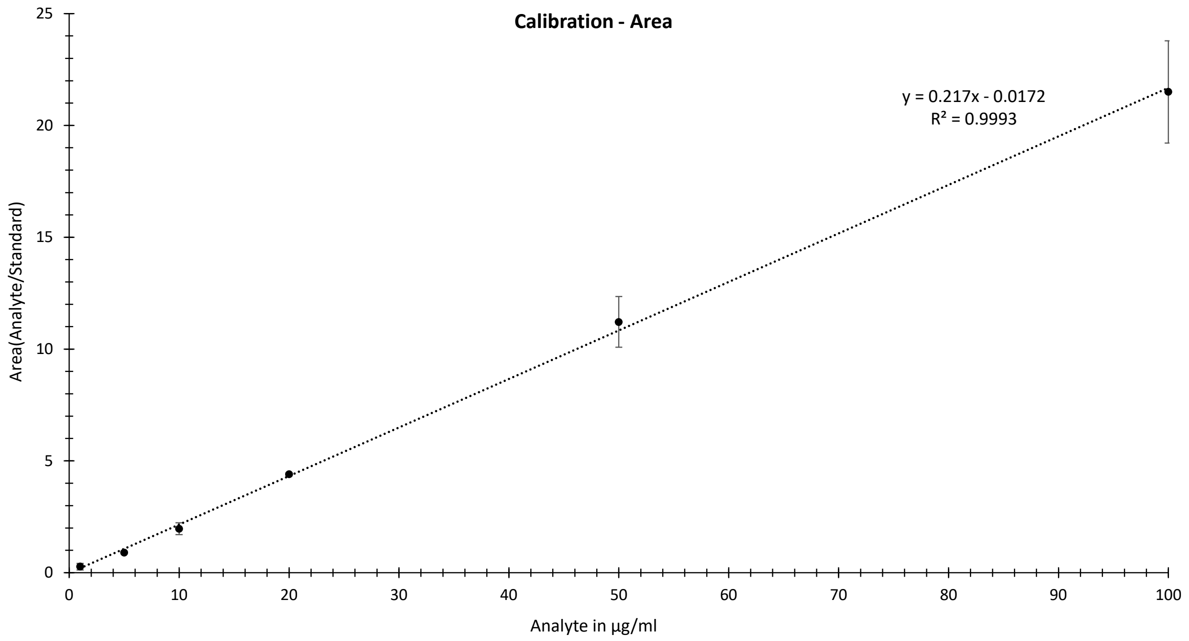

Figure S6: Calibration curve of isobaric pair 1 via MRM TIC area. (see Figure 4)

Table S5: Raw data for creation of calibration curve in Figure S4 via MRM TIC area (An = analyte; IS = internal standard).

Calibration I - Area								
Measurement	c(An)	A(An)	X̄(An)	A(IS)	X̄(IS)	ratio[A(An)/A(IS)]	X̄[A(An)/A(IS)]	σ[A(An)/A(IS)]
1	1 µg/ml	109182	104207.6	205609	446840	0.531017611	0.272369817	0.147154383
2		106338		648002		0.164101345		
3		105162		507913		0.20704727		
4		100292		451075		0.222339966		
5		100064		421601		0.237342891		
1	5 µg/ml	401332	411919.2	497189	456910	0.80720209	0.903747505	0.055320398
2		410811		452577		0.907715151		
3		419650		450533		0.931452302		
4		414047		444193		0.932133104		
5		413756		440056		0.940234879		
1	10 µg/ml	759033	772196.6	502451	399379	1.510660741	1.964864987	0.26400505
2		776828		397018		1.956656877		
3		784070		374830		2.091801617		
4		783000		366380		2.137125389		
5		758052		356214		2.128080311		
1	20 µg/ml	1314852	1320227	296480	300086	4.434875877	4.400322135	0.059931782
2		1323744		296061		4.471186681		
3		1314994		298234		4.409269232		
4		1311572		300075		4.370813963		
5		1335973		309578		4.315464923		
1	50 µg/ml	2598585	2640745.2	234620	237599	11.07571818	11.21233832	1.134267506
2		2656863		251891		10.54766943		
3		2678581		273823		9.7821622		
4		2633155		218454		12.05359023		
5		2636542		209207		12.60255154		
1	100 µg/ml	2858083	3052872.4	144820	142145	19.73541638	21.63515943	2.285727571
2		3054761		164088		18.61660207		
3		3120077		135443		23.03608898		
4		3122559		132795		23.5141308		
5		3108882		133580		23.27355892		

4. Method Qualification – Sensitivity

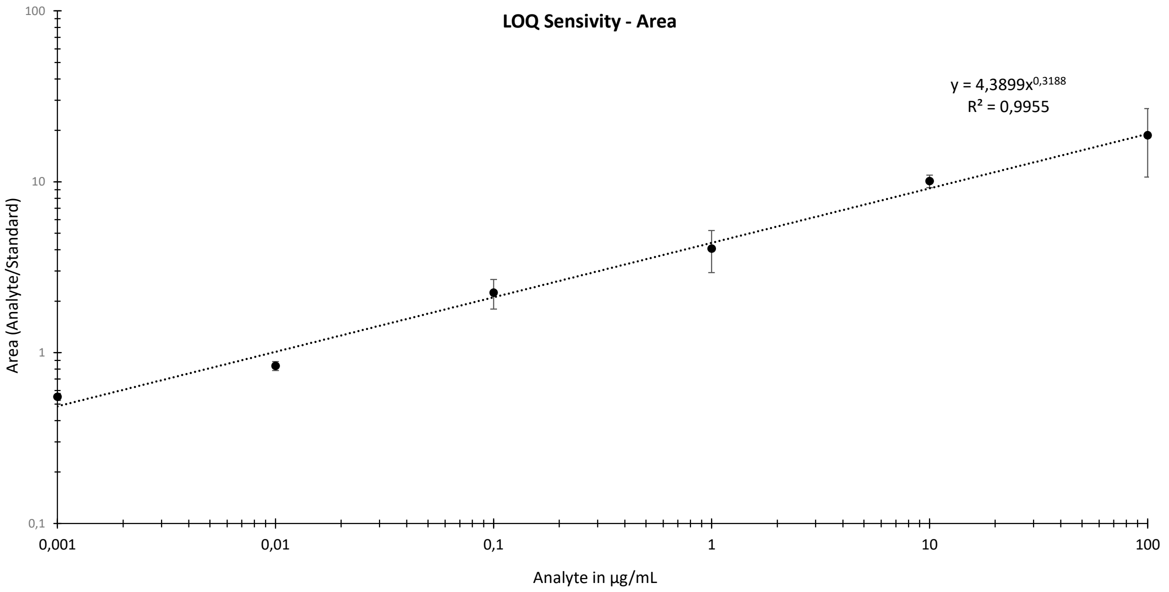

Figure S7: Sensitivity experiments of isobaric pair 1 analyte via MRM TIC area on a logarithmic scale. With LLOQ of 1 ng/ml.

Table S6: Raw data for sensitivity experiment in Figure S5 via MRM TIC area (An = analyte; IS = internal standard).

LOQ Sensitivity - Area								
Measurement	c(An/IS)	A(An)	X̄(An)	A(IS)	X̄(An)	ratio[A(An)/A(IS)]	X̄[A(An)/A(IS)]	σ[A(An)/A(IS)]
1		28072		48313		0,581044439		
2	1 ng/ml	26189	29049,33333	48823	52809	0,536407021	0,551340966	0,025724094
3		32887		61291		0,536571438		
1		50271		63633		0,790014615		
2	10 ng/ml	50121	50036	60427	60001	0,829447101	0,83603941	0,049650276
3		49715		55944		0,888656514		
1		89992		49595		1,814537756		
2	100 ng/ml	90803	91978	41026	41990	2,213303759	2,239763215	0,43905356
3		95140		35349		2,691448131		
1		167267		48518		3,44752463		
2	1 µg/ml	167966	170100	49699	43635	3,379665587	4,060907553	1,121691319
3		175067		32689		5,355532442		
1		288514		31548		9,145239001		
2	10 µg/ml	294439	292386	28621	29127	10,28751616	10,08156874	0,852228267
3		294204		27211		10,81195105		
1		411756		41109		10,01620083		
2	100 µg/ml	445799	434461	22030	26778	20,23599637	18,72715829	8,063122706
3		445828		17194		25,92927765		

Table S7: Raw data for precision determination of LLOQ in Figure S5 via MRM TIC area (An = analyte; IS = internal standard).

LLOQ Precision - Area								
Measurement	c(An/IS)	A(An)	X̄(An)	A(IS)	X̄(An)	ratio[A(An)/A(IS)]	X̄[A(An)/A(IS)]	σ[A(An)/A(IS)]
1		28072		48313		0,581044439		
2	1 ng/ml	26189	29049,33333	48823	52809	0,536407021	0,551340966	0,025724094
3		32887		61291		0,536571438		
1		27635		44525		0,620662549		
2	1 ng/ml	21763	26462	49963	49382	0,435582331	0,538372581	0,094227745
3		29988		53658		0,558872861		
1		25812		48655		0,530510739		
2	1 ng/ml	23658	28367,33333	51247	55255	0,461646535	0,511053017	0,043107607
3		35632		65863		0,541001776		

5. Method Qualification – Accuracy & Precision

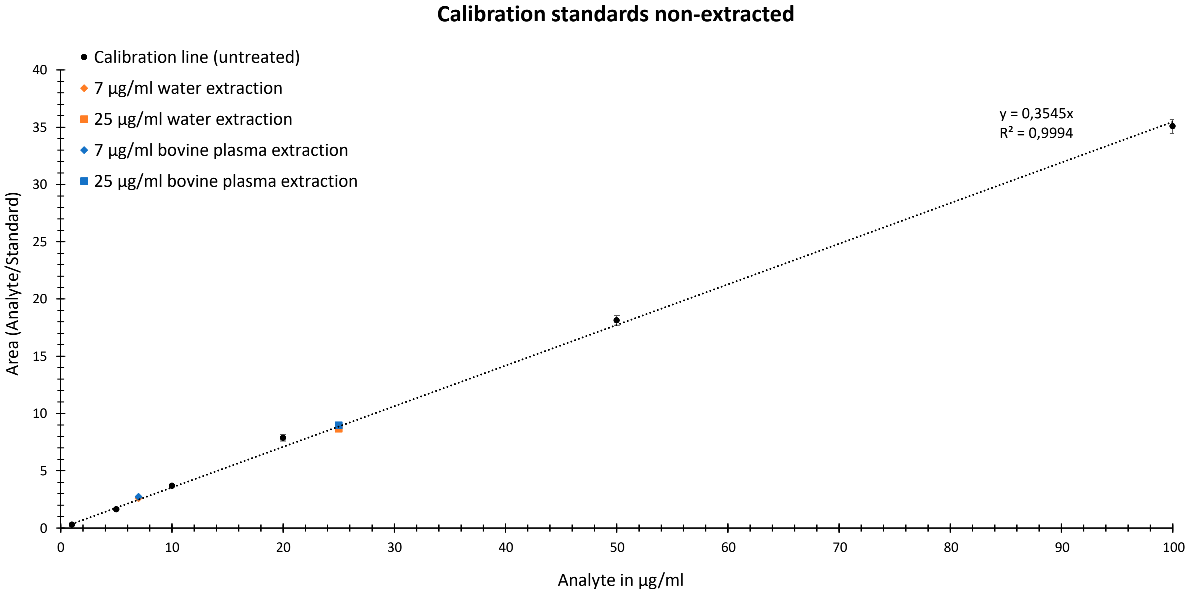

Figure S8: Calibration curve of isobaric pair 1 via MRM TIC area; non-extracted. (see Figure 5)

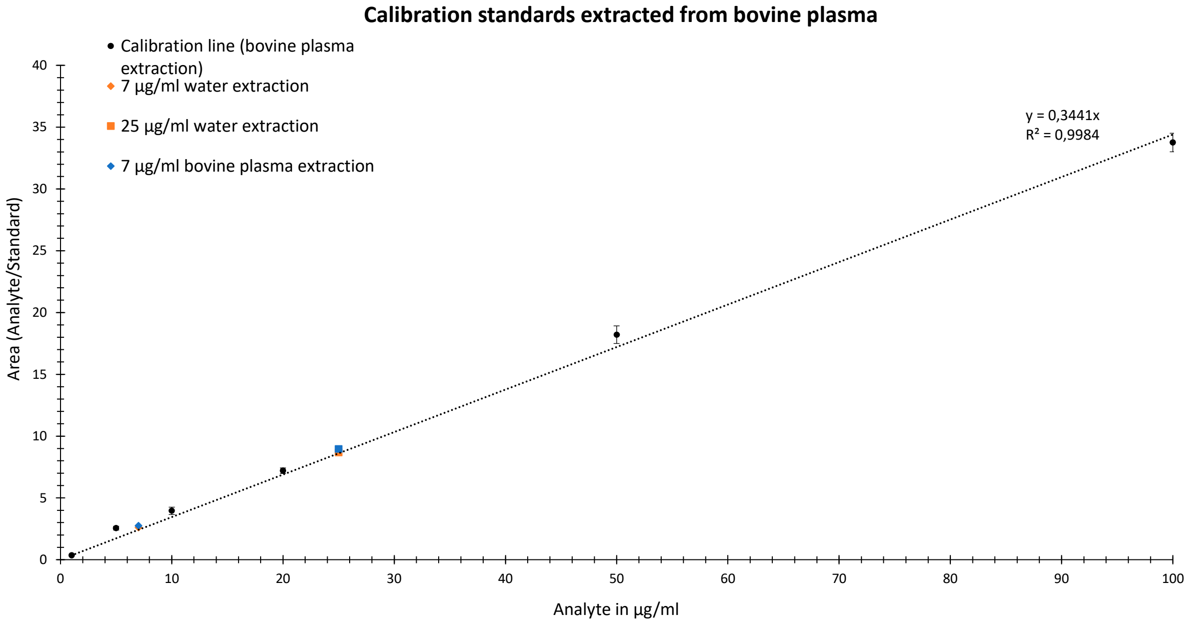

Figure S9: Calibration curve of isobaric pair 1 via MRM TIC area; extracted from bovine plasma. (see Figure 5)

Table S8: Raw data for non-extracted calibration curve in Figure S6 via MRM TIC area (An = analyte; IS = internal standard).

Calibration curve non-extracted - Area								
Measurement	c(An)	A(An)	X̄(An)	A(IS)	X̄(IS)	ratio[A(An)/A(IS)]	X̄[A(An)/A(IS)]	σ[A(An)/A(IS)]
1		1765		5938		0,297238127		
2	1 µg/ml	1677	1710	5716	5785	0,293386984	0,295571172	0,001976986
3		1688		5701		0,296088406		
1		7342		4433		1,656214753		
2	5 µg/ml	7708	7534	4629	4602	1,665154461	1,637940416	0,039646869
3		7553		4743		1,592452035		
1		14368		3805		3,7760841		
2	10 µg/ml	14162	14222	3899	3854	3,632213388	3,691193796	0,075353749
3		14137		3857		3,665283899		
1		31917		4162		7,668668909		
2	20 µg/ml	32466	32176	3966	4088	8,186081694	7,874877387	0,27422088
3		32144		4137		7,769881557		
1		77254		4344		17,78406998		
2	50 µg/ml	72547	74989	3902	4139,666667	18,59226038	18,12967702	0,416599958
3		75167		4173		18,01270069		
1		139568		3934		35,47737672		
2	100 µg/ml	140571	140361	4089	4002	34,37784299	35,08062098	0,610312064
3		140945		3983		35,38664323		

Table S9: Raw data for calibration curve extracted from bovine plasma in Figure S7 via MRM TIC area (An = analyte; IS = internal standard).

Calibration curve extracted from bovine plasma - Area								
Measurement	c(An)	A(An)	X̄(An)	A(IS)	X̄(IS)	ratio[A(An)/A(IS)]	X̄[A(An)/A(IS)]	σ[A(An)/A(IS)]
1		195		581		0,335628227		
2	1 µg/ml	195	195,33	596	560,6666667	0,327181208	0,350309416	0,033015161
3		196		505		0,388118812		
1		1577		584		2,700342466		
2	5 µg/ml	1414	1502,00	554	590	2,55234657	2,549946978	0,151609526
3		1515		632		2,397151899		
1		1883		520		3,621153846		
2	10 µg/ml	1914	1935,33	466	489,6666667	4,107296137	3,962623424	0,29686754
3		2009		483		4,15942029		
1		5385		729		7,386831276		
2	20 µg/ml	5353	5322,67	737	739,6666667	7,263229308	7,198537238	0,227641912
3		5230		753		6,945551129		
1		14583		788		18,50634518		
2	50 µg/ml	15290	14766,67	817	811,6666667	18,71481028	18,20102772	0,716978415
3		14427		830		17,38192771		
1		57220		1667		34,32513497		
2	100 µg/ml	57209	57570,00	1681	1706,333333	34,03271862	33,75545995	0,747896612
3		58281		1771		32,90852626		

Table S10: Raw data for sample testing extracted from water in Figure S6 & S7 via MRM TIC area (An = analyte; IS = internal standard).

Extraction from water - 7 µg/ml - Area								
Measurement	c(An)	A(An)	X̄(An)	A(IS)	X̄(IS)	ratio[A(An)/A(IS)]	X̄[A(An)/A(IS)]	σ[A(An)/A(IS)]
1		6023		2028		2,969921105		
2	7 µg/mL	5023	5305,333333	1969	2040	2,551041138	2,605322318	0,340716699
3		4870		2122		2,295004713		
**Extraction from water - 25 µg/ml - Area**								
Measurement	c(An)	A(An)	X̄(An)	A(IS)	X̄(IS)	ratio[A(An)/A(IS)]	X̄[A(An)/A(IS)]	σ[A(An)/A(IS)]
1		18127		1981		9,150429076		
2	25 µg/mL	17751	17660,66667	2019	2040	8,791976226	8,671379084	0,549367478
3		17104		2119		8,071731949		

Table S11: Raw data for sample testing extracted from bovine plasma in Figure S6 & S7 via MRM TIC area (An = analyte; IS = internal standard).

Extraction from bovine plasma - 7 µg/ml - Area								
Messung	c(An)	A(An)	X̄(An)	A(IS)	X̄(IS)	ratio[A(An)/A(IS)]	X̄[A(An)/A(IS)]	σ[A(An)/A(IS)]
1		6862		3267		2,100397919		
2	7 µg/mL	7411	7816,666667	2966	2923	2,498651382	2,739246718	0,787221661
3		9177		2536		3,618690852		
**Extraction from bovine plasma - 25 µg/ml - Area**								
Messung	c(An)	A(An)	X̄(An)	A(IS)	X̄(IS)	ratio[A(An)/A(IS)]	X̄[A(An)/A(IS)]	σ[A(An)/A(IS)]
1		37301		4330		8,614549654		
2	25 µg/mL	36915	36942,33333	4227	4131	8,733144074	8,963087554	0,504476649
3		36611		3837		9,541568934		

Table S12: Raw data for precision calculation by triplicate replica from analyte sample testing

Precision			
Calibration line (untreated)		Calibration line (Plasma extracted)	
7 µg/ml	25 µg/ml	7 µg/ml	25 µg/ml
2,605	8,6713	2,739	8,963
2,545	8,81	2,699	8,866
2,666	8,556	2,743	8,799

6. Method Qualification – Short term biofluid stability study

Table S13: Raw data for biofluid stability study in bovine plasma via MRM TIC area; set 1 (An = analyte; IS = internal standard).

Pharmacokinetic - Area - Bio. Rep. Set 1								
Measurement	t [h]	A(An)	X̄(An)	A(IS)	X̄(IS)	ratio[A(An)/A(IS)]	X̄[A(An)/A(IS)]	σ([A(An)/A(IS)]
1		60270		11587		5,201518944		
2	0	55014	56617	10659	10827	5,161272164	5,231400977	0,088918939
3		54567		10235		5,331411822		
1		51050		11885		4,295330248		
2	0,25	37744	45951	8117	10455	4,64999384	4,42082198	0,198768062
3		49060		11364		4,317141851		
1		38159		11069		3,447375553		
2	1	38721	38350	11686	11423	3,313451994	3,358669688	0,0768264
3		38171		11514		3,315181518		
1		21106		7798		2,706591434		
2	4	18255	20115	7094	7696	2,573301381	2,613243927	0,081121063
3		20983		8197		2,559838965		
1		37662		13603		2,768653973		
2	24	38645	38253	14616	13844,6667	2,644020252	2,766873384	0,121972585
3		38453		13315		2,887945926		
1		169835		64859		2,618526342		
2	48	172585	171369	65286	64827	2,643522348	2,643554487	0,025044231
3		171688		64336		2,668614772		
1		40551		18947		2,140233282		
2	96	38850	38918	18653	18561	2,082774889	2,096198211	0,039091893
3		37352		18083		2,065586462		
1		21556		14397		1,497256373		
2	192	21640	21534	15304	14872	1,414009409	1,4487994	0,043273595
3		21405		14915		1,435132417		

Table S14: Raw data for biofluid stability study in bovine plasma via MRM TIC area; set 2 (An = analyte; IS = internal standard).

Pharmacokinetic - Area - Bio. Rep. Set 2								
**Measurement**	**t [h]**	**A(An)**	**X̄(An)**	**A(IS)**	**X̄(IS)**	**ratio[A(An)/A(IS)]**	**X̄[A(An)/A(IS)]**	**σ[A(An)/A(IS)]**
1		53673		9979		5,37859505		
2	0	52394	54197,33333	9621	10090	5,445795655	5,373984655	0,074223665
3		56525		10670		5,297563261		
1		56826		11975		4,745386221		
2	0,25	54523	54119	12088	11534	4,510506287	4,698791524	0,169850825
3		51009		10538		4,840482065		
1		29997		9061		3,310561748		
2	1	28886	29201	8947	8893	3,228568235	3,283684472	0,047736916
3		28721		8672		3,311923432		
1		30447		10182		2,990276959		
2	4	28956	29805	10364	10430	2,793901968	2,859183991	0,113530149
3		30012		10744		2,793373045		
1		168859		51230		3,296096037		
2	24	171062	170655	53365	53122	3,205509229	3,214252202	0,077841471
3		172044		54771		3,141151339		
1		185730		69861		2,658564865		
2	48	45535	107349	17343	49781,6667	2,625554979	2,248335727	0,682150012
3		90781		62141		1,460887337		
1		40427		18899		2,139107889		
2	96	39652	39754	18587	18647	2,133318986	2,131863777	0,008070719
3		39183		18455		2,123164454		
1		25486		16009		1,591979512		
2	192	22662	24156	13708	14778	1,653195214	1,636330277	0,038774265
3		24320		14617		1,663816105		

Table S15: Raw data for biofluid stability study in bovine plasma via MRM TIC area; set 3 (An = analyte; IS = internal standard).

Pharmacokinetic - Area - Bio. Rep. Set 3								
**Measurement**	**t [h]**	**A(An)**	**X̄(An)**	**A(IS)**	**X̄(IS)**	**ratio[A(An)/A(IS)]**	**X̄[A(An)/A(IS)]**	**σ[A(An)/A(IS)]**
1		56939		11294		5,041526474		
2	0	62963	59751	12208	11701,66667	5,157519659	5,10472959	0,058693512
3		59351		11603		5,115142636		
1		48366		11243		4,301876723		
2	0,25	53072	51104	12341	11828	4,300461875	4,320499421	0,033488226
3		51874		11900		4,359159664		
1		187649		55191		3,399992752		
2	1	192393	191459	56560	56081	3,40157355	3,413844105	0,02263604
3		194334		56493		3,439966013		
1		29244		9748		3		
2	4	27458	28074	9829	9584	2,793570048	2,931081012	0,119088101
3		27519		9174		2,999672989		
1		248305		84560		2,936435667		
2	24	87762	191901	64909	77012,66667	1,352077524	2,408781655	0,915132888
3		239636		81569		2,937831774		
1		125391		45830		2,736002618		
2	48	117914	119164	43008	42991,33333	2,741675967	2,774235165	0,061373039
3		114188		40136		2,845026909		
1		38665		22204		1,741352909		
2	96	42176	39595	25987	23122,33333	1,622965329	1,718735093	0,086702425
3		37945		21176		1,791887042		
1		33653		18553		1,813884547		
2	192	30429	31566	16115	17203,33333	1,888240769	1,836390964	0,045033186
3		30615		16942		1,807047574		

Table S16: Raw data for calibration curve for biofluid stability study in bovine plasma (An = analyte; IS = internal standard).

Calibration for Biofluid stability study - Area								
**Messung**	**c(An)**	**A(An)**	**X̄(An)**	**A(IS)**	**X̄(IS)**	**ratio(An/IS)**	**X̄(An/IS)**	**σ(An/IS)**
1		14960		122563		0.122059675		
2	1 µg/ml	14846	14545	120747	123027.667	0.122951295	0.11832101	0.00726142
3		13829		125773		0.109952056		
1		49646		118456		0.419109205		
2	5 µg/ml	48255	48938	118937	111375	0.405718994	0.44349778	0.0542532
3		48914		96732		0.505665137		
1		94084		81805		1.15010085		
2	10 µg/ml	94984	94323	118846	98795	0.799219158	0.9767175	0.17547704
3		93900		95735		0.980832506		
1		165417		70232		2.355293883		
2	20 µg/ml	165518	159832	71650	71029	2.310090719	2.25059475	0.14398214
3		148560		71204		2.08639964		
1		386639		77905		4.962954881		
2	50 µg/ml	398394	395840	76688	76264.6667	5.194997914	5.1940731	0.23065721
3		402486		74201		5.424266519		
1		688096		68268		10.07933439		
2	100 µg/ml	688428	686501	62120	66317	11.08222795	10.3742991	0.61591676
3		682979		68563		9.961334831		

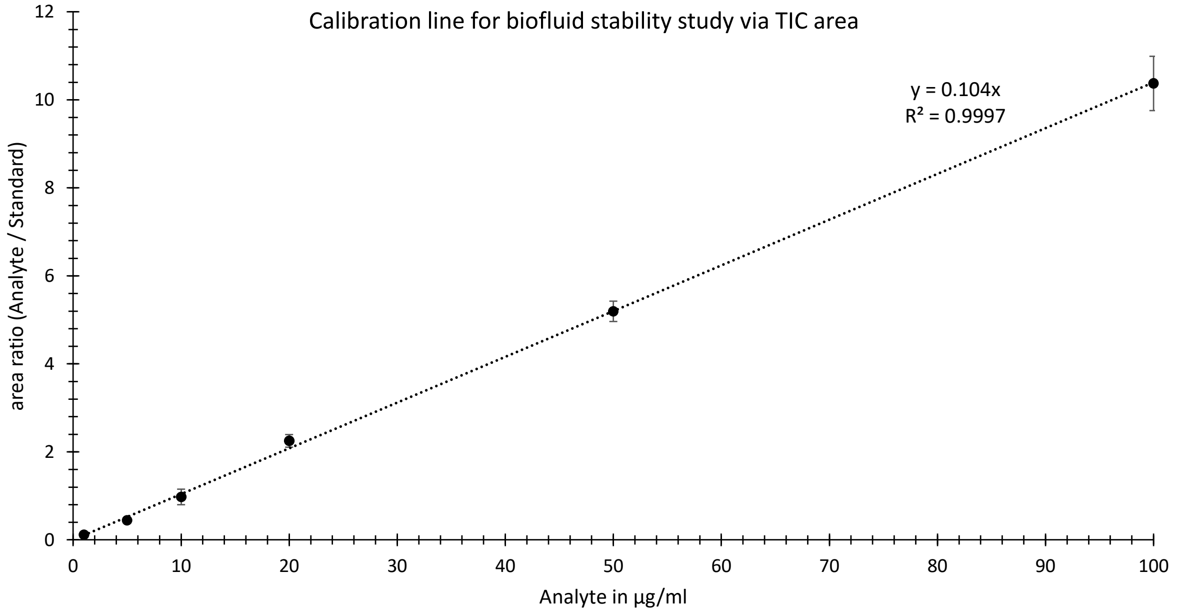

Figure S10: Calibration curve for biofluid stability study from raw data in table S16.

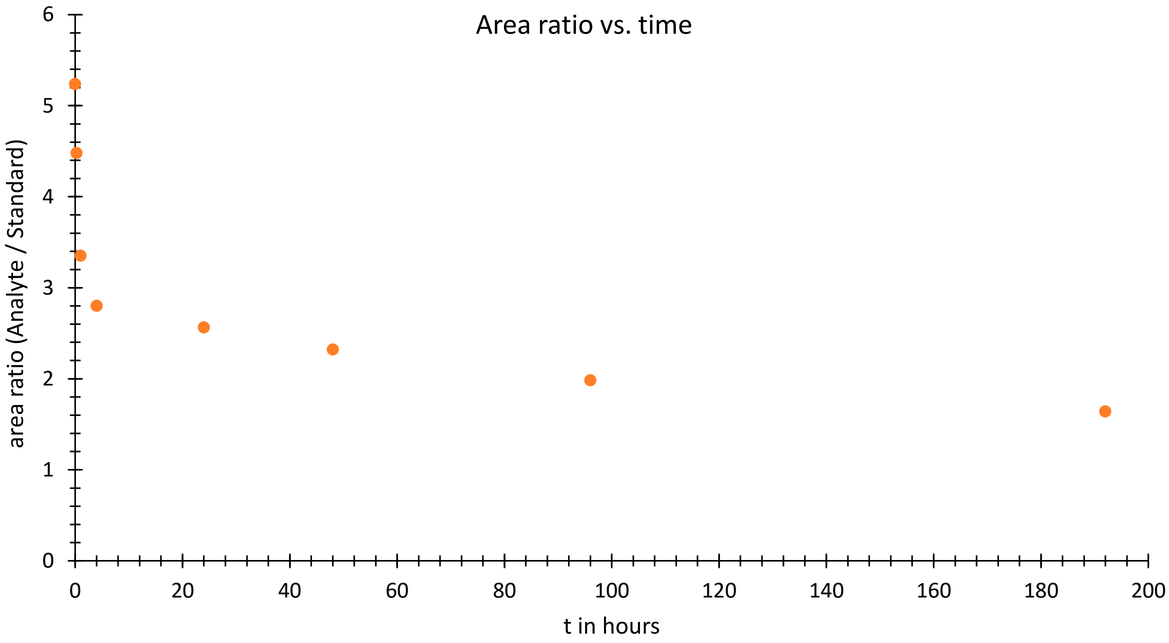

Figure S11: Mean area ratios in relation to degradation time in hours.
